# The Reciprocal Relationship between Socioeconomic Status and Health and the Influence of Sex: A European SHARE-Analysis Based on Structural Equation Modeling

**DOI:** 10.3390/ijerph18095045

**Published:** 2021-05-10

**Authors:** Linda Juel Ahrenfeldt, Sören Möller

**Affiliations:** 1 Unit for Epidemiology, Biostatistics and Biodemography, Department of Public Health, University of Southern Denmark, 5000 Odense, Denmark; 2 OPEN–Open Patient Data Explorative Network, Odense University Hospital, 5000 Odense, Denmark; moeller@health.sdu.dk; 3 Department of Clinical Research, University of Southern Denmark, 5000 Odense, Denmark

**Keywords:** structural equation modeling, bidirectional, reciprocal relationship, sex differences, income, wealth, cognitive function, grip strength, quality of life, depressive symptoms

## Abstract

It is well recognized that socioeconomic status (SES) is an important determinant of health, but many studies fail to address the possibility of reverse causation. We aim to investigate the reciprocal relationship between trajectories of SES and health, and how these associations differ by sex. We performed a longitudinal study including 29,824 men and 37,263 women aged 50+ participating in at least two consecutive waves of the Survey of Health, Ageing and Retirement in Europe (SHARE). Using structural equation modeling, we found that baseline household income and wealth led to improvements in cognitive function, grip strength, quality of life and depressive symptoms, and a better initial health led to higher income and wealth for both sexes. However, the results indicated that the relative effect of cognitive function and grip strength on SES trajectories was overall greater than the corresponding effect of SES on health changes, particularly regarding income among women, but for quality of life and depressive symptoms, the reverse was indicated, though most pronounced for the associations with wealth. The reciprocal associations between SES and physical function were stronger for men than for women, whereas most associations with cognitive function and mental health were similar between sexes. This study demonstrates that both social causation and health selection contribute to social inequalities in health, but the influence of each direction and the importance of sex differences may vary according to the health outcomes investigated.

## 1. Introduction

Differentials in health and mortality by socioeconomic status (SES) have been identified by numerous studies in medical and social sciences, some of which date back to the 1800s [1]. With a few exceptions, these studies have found that people with higher SES experience lower rates of morbidity and mortality compared with their better-off counterparts. These health inequalities have been identified across age, sex, time, and place. They cover a broad set of outcome variables such as self-rated health, mental and physical diseases, disability, and mortality and are apparent for a wide range of SES measures such as education, income, occupation, wealth, and combinations thereof [1,2,3,4,5,6].

Different explanations for the observed patterns between SES and health have been proposed [7]. The social causation hypothesis relates to a set of causal mechanisms through which SES affects health, e.g., the experience of adversity and stressors in low social status groups [8]. This explanation has received empirical support in a variety of contexts [1,7]. The health selection hypothesis (also known as reverse causation), however, states that individuals’ health influences their ability to attain and maintain desirable socioeconomic positions, so unhealthy individuals may reduce their social position as a consequence of their inferior health status [1,7]. Overall, the evidence regarding the social causation and health selection hypotheses is mixed and differs by the way in which SES and health are measured and by the methodology utilized [7]. 

Some studies using structural equation modeling (SEM) have recognized that both causal directions between SES and health may have merit [9,10,11,12,13]. For example, a longitudinal study of 2976 participants aged 31–47 years followed for 16 years from the annual Swedish Survey of Living Conditions found that initial health affects occupational mobility, and that more prestigious jobs are related to initially good health and to a less rapid deterioration in health. Moreover, it was demonstrated that change in occupation and income was related to health change [11]. A study based on the Wisconsin Longitudinal Study of 10,317 white high school graduates in 1957, who were employed in 1993, investigated the reciprocal relationships between perceived social position (participants’ judgment of their social position) and health outcomes (i.e., self-reported health, the Health Utilities Index, and depressive symptoms). The study found that the relationship differed across operationalizations of perceived social position and health outcomes, but indicated that the causal relationship of perceived social position affecting health did not necessarily hold in empirical models of reciprocal relationships [12]. Based on data from the Whitehall II study [10], Chandola et al. found effects of mental health on changes in financial deprivation (a measure of social position) among men, although this health selection effect was two and a half times smaller than the effect of social position on health changes, lending most support for the social causation hypothesis. Similar findings of the stronger causal path from SES to health were observed by Mulatu and Schooler in a sample of 707 men and women, mainly European Americans, with a mean age of 64 years. They also found that about one third of the overall SES–health relationship was accounted for by health-related lifestyles/behaviors such as sleep, weight, and psychological distress [9]. 

Despite living longer than men, women systematically report higher rates of morbidity, disability, and healthcare utilization, and they perform worse on physical tests [14,15,16,17,18,19,20]. Research has, so far, highlighted explanations for sex differences in health including biological, psychosocial, behavioral, and social factors [18,21], with SES widely recognized as the most important determinant of sex differences in health [16,18]. Some evidence shows that SES gradients in mortality are generally weaker among women than among men [22,23,24,25]. A recent population-based registry study including one million Danes investigated sex differences in mortality and hospitalizations by income trajectories. Overall, the study found that income has a larger influence on men’s than women’s health and mortality, and that income in the late 50s is an important predictor of mortality, particularly among men [26]. However, it is still unclear to what extent SES has the same differential impact on the health of women and men in later life. Most of the existing evidence is from single-country cross-sectional studies, and, overall, studies have reported mixed results depending on the SES indicator and the health outcome considered [27]. Moreover, researchers have rarely utilized analytical techniques that allow them to estimate models of reverse causation in which SES and health are hypothesized to affect one another simultaneously [7].

Here we aim to examine the reciprocal relationship between trajectories of SES (i.e., household income and wealth) and health, and how these associations differ by sex. Thus, using SEM, we assess the influence of SES on health changes among middle-aged and older Europeans, and, similarly, we evaluate a concurrent influence of health on trajectories of income and wealth. We hypothesize that there is a reverse association between trajectories of SES and health, but that the influence of SES on health changes is greater than the reverse. Furthermore, we hypothesize that the reciprocal associations with physical health are stronger for men than for women, and that the associations with cognitive function and mental health may be stronger for women than for men. 

## 2. Materials and Methods

### 2.1. Setting and Study Participants

The Survey of Health, Ageing and Retirement in Europe (SHARE) is a multinational panel study collecting individual-level data on health, social, and economic factors in Europeans aged 50 and older and their spouses/partners at any age. SHARE has been conducted biannually since 2004 and operates in 27 European countries and Israel [28,29]. The samples in SHARE are drawn at the household level. Most data are collected as computer-assisted personal interviews at the participants’ homes by trained interviewers with questionnaires strictly harmonized across countries [29]. Respondents who were interviewed in a previous wave are part of the longitudinal sample. To compensate for attrition and to maintain representation of the younger age cohorts, refreshment samples are drawn regularly [28]. The household response rate (i.e., the proportion of selected households including at least one interview) differed by country and wave, varying between 44.0% and 97.5% in wave 1 and between 35.2% and 84.3% for refreshers in wave 7 [28]. If a respondent in SHARE is not able to complete the interview due to, e.g., physical or cognitive limitations, a proxy respondent (any person of the close social network, e.g., a family member) is allowed to help; however, some questionnaire modules cannot be answered by other persons, such as the cognitive function section [30]. 

The current study included respondents aged 50 and older who participated in at least two consecutive waves of SHARE in 2004–05 (wave 1), 2006–07 (wave 2), 2011 (wave 4), 2013 (wave 5), 2015 (wave 6) or 2017 (wave 7). We excluded the third wave (2008–09, SHARELIFE) because it focused on respondents’ retrospective life histories, hence handling waves 2 and 4 as being consecutive waves. Moreover, we included only individuals from wave 7 who had completed a regular interview (i.e., those who did not receive the SHARELIFE questionnaire). 

### 2.2. Socioeconomic Status

SES was estimated by the total household net income and household net worth (wealth). Income is obtained by an aggregation at the household level of all individual income components. Wealth is the sum of household net financial assets and household real assets [30]. Income and wealth were calculated based on an average of the provided imputations for each wave of SHARE, which compensates for nonresponse. The imputed SES components were an integral part of the original SHARE dataset. The average income and wealth in each wave were divided into deciles for men and women separately, which formed the basis for the trajectories. 

### 2.3. Health Variables

We used performance-based measures on cognitive and physical functioning. Cognitive function was tested by three cognitive tasks: (1) fluency—the number of animals that the participant could name in one minute, (2) immediate recall—measuring how many of ten words the respondent could recall immediately after the interviewer read the words, (3) delayed memory—measuring the ability to recall the same words after other interview questions. In line with previous research [20,31,32], we calculated a cognitive composite score (CCS) by standardizing each of the three tests to the mean and standard deviation (SD) of the youngest age group (i.e., the 50–54 year-olds) in the total study population, before summing them into the CCS. The CCS was linearly transformed into a T-score with a mean of 50 and an SD of 10 in the youngest age group, with higher scores reflecting better performance. If a person had missing information in one or more of the tests, the CCS was coded as missing. Physical function was proxied by grip strength, measured as the maximum score in kilograms out of four trials including two measurements per hand, recorded with a handheld dynamometer, as described in detail previously [33]. 

Quality of life and depressive symptoms were subjectively measured variables. Quality of life was investigated by the CASP-12 index, a 12-item self-assessed questionnaire. The scale is composed of four subscales: control, autonomy, self-realization, and pleasure. The 12 items are assessed on a four-point Likert scale (often, sometimes, rarely, and never). The resulting score is the sum of these 12 items and ranges from 12 to 48, with higher scores meaning better quality of life [34]. Depressive symptoms were measured on the Euro-depression (EURO-D) 12-item scale [35]. The tool assesses 12 items (depressed mood, pessimism, wishing death, guilt, sleep, interest, irritability, appetite, fatigue, concentration, enjoyment, and tearfulness), and each symptom is scored with one point. The sum creates a scale ranging from 0 to 12 [34]. We reversed the scale so 12 was not depressed and 0 was very depressed. Thus, in line with the other health measures, higher scores reflected better health. 

### 2.4. Socio-Demographic Variables

Demographic variables included sex, age at interview, wave, European region, marital status, and employment. Age was grouped into 5-year categories from age 50 to 90, with an open-ended category from age 90 years. Sixteen European countries were included in at least two consecutive waves of SHARE. In accordance with previous studies [14,15,20], these countries were categorized into four regions: Northern Europe (Denmark and Sweden), Western Europe (Austria, Germany, France, the Netherlands, Switzerland, Belgium, and Luxembourg), Southern Europe (Spain, Italy, and Greece) and Eastern Europe (Czech Republic, Poland, Slovenia, and Estonia). Marital status was grouped into married/registered partnership, unmarried/divorced and widowed. Employment was categorized into employed/self-employed including homemakers, unemployed/sick, and retired. 

### 2.5. Statistical Methods

**Figure 1:**
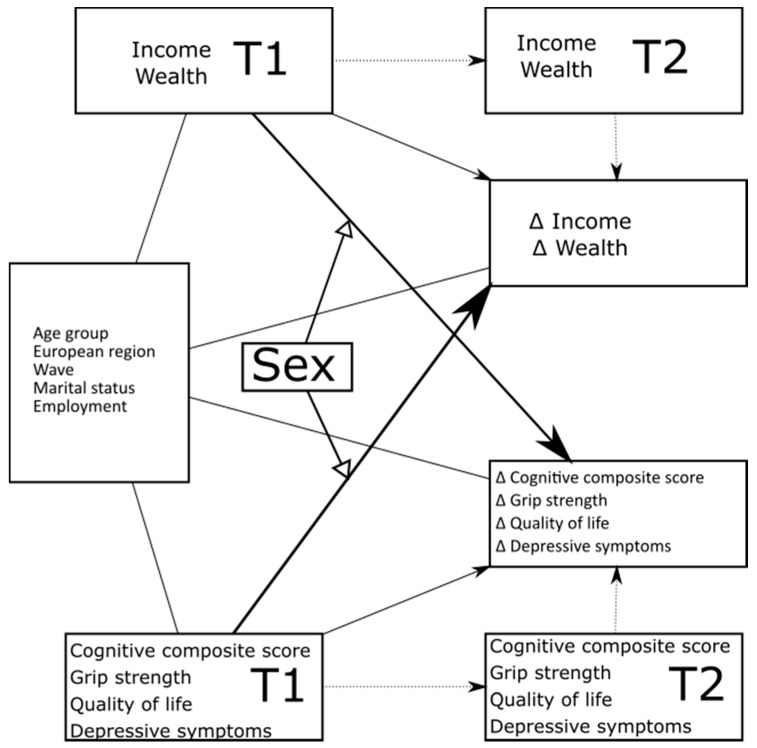
Illustration of the structural equation models used to investigate the dynamic interplay between trajectories of SES and health over time. T1: time point 1, T2: time point 2.

We used a change score approach including a series of SEM models to investigate the dynamic interplay between trajectories of SES and health over time reporting standardized coefficients with 95% confidence intervals (CIs). We constructed a model including all pairs of consecutive waves in the dataset, investigating the associations between SES at time point 1 (T1) and health changes from T1 to time point 2 (T2), and vice versa between initial health at T1 and SES trajectories from T1 to T2 (Figure 1). The coefficient reported the measure of the difference in the dependent variable (the change from T1 to T2) per one-unit change in the independent variable (measurement at T1). Thus, for instance, a coefficient of 0.1 for the association between income at T1 and change in cognitive function between T1 and T2 implies that a person with a one SD higher income at T1 would on average show an increase of 0.1 SD in cognitive function. We considered repeated measurements from the same individual by clustered robust standard errors, hereby taking deviations from the independence assumption into account.

We constructed three models: (1) a crude model, (2) a model adjusted for age, wave, European region, and marital status, (3) a model further adjusted for employment. All models were investigated separately for men and women. In a subsequent analysis, we examined whether the associations differed by sex. In the examination of sex differences, we applied bootstrapping with 1000 repetitions to compensate for deviations from normality assumptions of residuals. Differences between the reciprocal associations were investigated by a Wald test. As our SEM models do not include any latent variables, no checks of the goodness of fit of the latent structure were performed. All analyses were performed in Stata version 16.0. 

## 3. Results 

In total, 67,087 participants were included in the study corresponding to 203,233 observations. The total sample had an overall mean age of 67.0 years (SD = 9.8), and 44.0% were men. Median income and wealth were higher among men than among women (Table 1). Compared with women, men had on average slightly lower CCS (men: mean = 44.7, SD = 10.7; women: mean = 45.9, SD = 11.6; *p* < 0.001), higher grip strength measures (men: mean = 42.8, SD = 10.1; women: mean = 26.3, SD = 6.9; *p* < 0.001), they reported better quality of life (men: mean = 38.0, SD = 6.0; women: mean = 37.2, SD = 6.4; *p* < 0.001), and had fewer depressive symptoms (men: mean = 10.1, SD = 2.0; women: mean = 9.2, SD = 2.3; *p* < 0.001) (Table 1). The reciprocal associations between trajectories of income and health and between trajectories of wealth and health are presented in Table 2 and Table 3. 

We found that income had a positive influence on change in cognitive function and grip strength for both women and men. Similarly, cognitive function and grip strength had a positive influence on the trajectories of income for both sexes (Table 2). When exa-mining differences between the reciprocal associations, we found that for women, the relative effect of cognitive function and grip strength on income trajectories was greater than the corresponding effect of income on health changes. This was the case in all the models investigated. For men, a significant difference was found in the crude model between income and cognitive function (Table 2). No sex differences were detected in most associations. However, the effect of cognitive function on income trajectories was stronger for women than for men (*p* = 0.028). Contrarily, the effect of income on improvements in grip strength was stronger for men than for women (*p* = 0.002) (Table 2). When investigating associations with wealth, we detected a similar pattern with reciprocal associations between wealth and health for both men and women, although less pronounced compared with the results for income (Table 3). In the crude models, we found that the effect of grip strength on wealth trajectories for both sexes and the effect of cognitive function on wealth trajectories for women were stronger than the reverse. In model 2 for men, however, a reverse pattern was indicated with the strongest effect of wealth on health changes. Regarding sex differences, we found that both the effect of wealth on change in grip strength (*p* = 0.003) and the effect of grip strength on wealth trajectories (*p* = 0.004) were stronger for men than for women (Table 3).

When we investigated the associations with quality of life and depressive symptoms, we found that income had a positive influence on change in quality of life and depressive symptoms for both men and women. Furthermore, quality of life and depressive symptoms had a positive influence on trajectories of income (Table 2). The results indicated that the effect of income on health changes was stronger than the reverse, with significant differences in the crude models for quality of life for both sexes and for depressive symptoms for men (Table 2). Overall, a similar pattern was found for wealth. The effect of wealth on health changes was significantly greater than the effect of health on wealth trajectories in all investigated associations (Table 3). No sex differences were found in any of the associations with quality of life and depressive symptoms (Table 2 and Table 3).

Overall, the strongest associations were found in the crude models. Adjusting for age, wave, European region, and marital status reduced the strength of the associations, and these were further reduced after also controlling for employment (Table 2 and Table 3).

In all the analyses, income had a negative association with income trajectories for both men and women, but with a stronger association for women than for men (*p* < 0.001). Furthermore, wealth had a negative association with wealth trajectories, but with overall similar associations for men and women (Supplementary Table S1). High cognitive function had a negative influence on change in cognitive function, but with the strongest association for women (*p* < 0.001). Likewise, high grip strength had a negative influence on change in grip strength, high quality of life had a negative influence on change in quality of life, and a number of depressive symptoms had a negative influence on change in depressive symptoms with similar associations for men and women (Supplementary Table S1).

## 4. Discussion

By use of SEM, we investigated the reciprocal associations between the SES of middle-aged and older adults and their cognitive, physical, and mental health, and how these associations differ between men and women. As hypothesized, we found a reciprocal association between trajectories of SES and health for both sexes. However, our findings indicated that the relative effect of cognitive function and grip strength on SES trajectories was somewhat greater than the corresponding effect of SES on health changes, mainly regarding income for women, whereas the reverse was suggested for quality of life and depressive symptoms, most pronounced for wealth. Overall, the reciprocal associations between SES and grip strength were stronger for men than for women, whereas no sex differences were found in most of the associations with cognitive function, quality of life and depressive symptoms.

In line with previous studies using SEM [9,10,11,12,13], our study indicates that both social causation and health selection contribute to social inequalities in health, but in agreement with the notion of previous researchers [7,12], we show that the relative merits of the social causation and health selection hypothesis vary across measures of health. We demonstrated, in accordance with findings from the Whitehall II study [10], that health selection was present, but that social causation was most pronounced when investigating associations with mental health outcomes. However, a recent study comparing the transitions from childhood to adulthood and from adulthood to old age showed that health-related social mobility was strongest in younger ages, whereas social causation was found to be most important in the transition between adulthood and old age [36]. Although our study only investigated SES–health associations in Europeans with an average age of 67 years, we found that health selection was stronger than social causation regarding the objectively measured health variables (cognitive and physical functioning), particularly among women. Another finding in the present study was that higher baseline SES was associated with less increase in SES, while better initial health prompts weaker improvements in health, most likely because higher baseline scores leave less room for a positive change [37]. Most of these associations were similar between sexes, although the association between initial cognitive function and change in cognitive function was stronger among women than among men.

We hypothesized that, for men, physical function would be more strongly associated with SES, whereas for women, the associations may be strongest with cognitive function, quality of life and mental health. In this study, only the influence of cognitive function on income trajectories was greater for women than for men. Thus, although SES has been widely recognized as one of the most important determinants of sex differences in health [16,18], results from this study do not support the notion that short-term changes in income and wealth among the middle-aged and elderly population can explain the poorer health of women compared with men, in line with evidence from Denmark [26]. A potential explanation could be that women, especially those in the included age cohorts, are not in society expected to provide financially for the household to the same degree as men. Hence, men’s physical strength might be more important for improving household income and wealth than that of women. Our results add support to the notion that, in relation to physical function, men benefit more from an increase in SES compared with women, and that grip strength has a greater influence on wealth for men than for women. There are different pathways through which wealth may relate to grip strength. Earlier evidence from SHARE showed that the association between wealth and grip strength was attenuated by about half after adjustment for chronic diseases and disability. The evidence suggests that the associations partly reflect the physical disability consequences of chronic conditions [38], which may be stronger for men than for women because men suffer more from diseases with high lethality such as stroke and heart conditions. Another possible mechanism involves the role of diet and nutrition [39]. Limited financial resources might reduce access to adequate diet and nutrition, and this may also be most pronounced for men due to the generally healthier nutrition of women [40]. A likely explanation for the association between grip strength and SES trajectories is that frail individuals may be less able to work and thus accumulate less income and wealth, and this relationship may be strongest among men because they often have more physically demanding jobs than women [41].

Earlier studies [26,42,43,44] have demonstrated smaller relative associations between individual income and mortality with advancing age, indicating that income best predicts differences in health and mortality among the youngest elderly. In the present study, we used household wealth as a measure of SES in addition to household income. Although income and wealth are both measures of financial well-being and consumption capability, income also reflects a flow of resources, available over a period, while wealth reflects resources accumulated over a lifetime [45]. Among the elderly, levels of wealth vary much more than levels of income and may thus allow more accurate measurements of SES differences in health [45]. Furthermore, evidence shows that the problem of reverse causation is more likely to influence household income than household wealth, mainly because wealth is less affected by a single episode of sickness [46]. In line, we found that the influence of health on income trajectories was larger than the influence of health on trajectories of wealth, though it was most pronounced for cognitive function and quality of life.

Labor force status is an important determinant of income and is likely to have an association with health independent of income. However, it is also a proxy for health status because people in poor health tend not to be active in the labor force [47]. Therefore, adjusting income–health associations for employment status may be problematic because it adjusts both for confounding by labor force status and for reverse causation [47]. Although further adjustment for employment slightly reduced the strength of the SES–health associations in the present study, significant associations were still found between SES and health for all investigated associations, suggesting that income and wealth have an independent effect on health.

The main strength of this study was the ability to apply SEM in a large sample of middle-aged and elderly Europeans from all waves of SHARE, allowing us to model the complex interplay between SES and health and to investigate how these associations are modified by sex over a 2–4-year period. To ensure sufficiently large numbers of observations regarding income and wealth and to compensate for non-response, we used the imputed values provided by SHARE. This increased the available sample size, but resulted in increased uncertainty. Requiring participation in two consecutive waves ensured comparability of the reciprocal associations, but made us only capable of capturing short-term effects. By using performance-based measures on cognitive and physical functioning, we avoided bias that may arise in self-reports, whereas misclassification may be an issue when using self-reported data. In this study, we investigated only associations with trajectories of SES and health, and we did not differentiate between levels of SES and health. If sex differences are most pronounced for people with low SES [26], there may be differences between men and women among the lower SES groups that we did not find in this study. Due to numerical challenges of fitting SEM on subsamples, we were not able to investigate associations between SES and health for the different European regions, which should be considered in future studies together with investigations of more long-term effects.

## 5. Conclusions

The results from SEM applied on a large national sample of Europeans aged 50 and older demonstrated that baseline household income and wealth were associated with improvements in cognitive function, grip strength, quality of life, and depressive symptoms, and a better initial health was associated with increases in income and wealth for both sexes. However, our findings indicated that the relative effect of cognitive function and grip strength on SES trajectories was somewhat greater than the corresponding effect of SES on health changes, mainly for women in relation to income, whereas the reverse was suggested for quality of life and depressive symptoms, most pronounced for wealth. Overall, the reciprocal associations between SES and physical function were stronger for men than for women, whereas only the influence of cognitive function on income trajectories was greater for women than for men. No sex differences were found in the associations with quality of life and depressive symptoms. This study demonstrates that both social causation and health selection contribute to social inequalities in health, but the influence of each direction and the importance of sex differences may vary according to the health outcomes investigated.

## Figures and Tables

**Table 1:** Baseline characteristics of 67,087 European men and women (corresponding to 203,233 observations) participating in at least two consecutive waves of the Survey of Health, Ageing and Retirement in Europe (SHARE).

	Men	Women
Individuals	29,824 (44.5)	37,263 (55.5)
Observations ^a^	89,500 (44.0)	113,733 (56.0)
European regions		
Northern Europe	12,948 (14.5)	15,237 (13.4)
Western Europe	39,645 (44.3)	49,069 (43.1)
Southern Europe	19,153 (21.4)	23,619 (20.8)
Eastern Europe	17,754 (19.8)	25,808 (22.7)
Age, mean (SD)	67.0 (9.6)	67.0 (10.1)
Marital status		
Married/registered partnership	70,648 (79.3)	69,306 (61.2)
Unmarried/divorced	11,634 (13.1)	16,568 (14.6)
Widowed	6776 (7.6)	27,452 (24.2)
Missing	442 (0.5)	407 (0.4)
Employment		
Employed/self-employed/homemakers	24,615 (27.8)	43,921 (39.3)
Unemployed/sick	5519 (6.2)	6075 (5.4)
Retired	58,443 (66.0)	61,688 (55.2)
Missing	923 (1.0)	2049 (1.8)
Socioeconomic status		
Income, median (IQR) in Euros	24,976 (12,600–46,200)	19,818 (9890–38,126)
Wealth, median (IQR) in Euros	170,088 (59,414–358,984)	138,434 (38,000–310,000)
Cognitive composite score		
Mean (SD)	44.7 (10.7)	45.9 (11.6)
Missing	3570 (4.0)	3638 (3.2)
Grip strength		
Mean (SD)	42.8 (10.1)	26.3 (6.9)
Missing	6080 (6.8)	10,040 (8.8)
Quality of life (12–48)		
Mean (SD)	38.0 (6.0)	37.2 (6.4)
Missing	7627 (8.5)	9603 (8.4)
Depressive symptoms (0–12)		
Mean (SD)	10.1 (2.0)	9.2 (2.3)
Missing	2952 (3.3)	3194 (2.8)

^a^ The rest of the table is given in observations. Data are numbers (percentages) unless stated otherwise. Percentages of categories are calculated without missing values. IQR: interquartile range, SD: standard deviation.

**Table 2:** Coefficients and 95% confidence intervals (CIs) for the associations between income and health changes (i.e., change (∆) in cognitive function, grip strength, quality of life and depressive symptoms) and for associations between health and trajectories (∆) of income.

	Men		Women		Sex Differences (Men vs. Women)
	Coefficients (95% CI)	*p*-Values ^a^	Coefficients (95% CI)	*p*-Values ^a^	*p*-Values ^b^
Cognitive composite score (CCS)					
*Model 1* ^c^					
Income → ∆ CCS	0.119 (0.111, 0.127)		0.122 (0.115, 0.129)		0.595
CCS → ∆ income	0.130 (0.122, 0.138)	0.028	0.142 (0.135, 0.148)	<0.001	0.023
*Model 2* ^d^					
Income → ∆ CCS	0.108 (0.098, 0.118)		0.099 (0.090, 0.109)		0.868
CCS → ∆ income	0.107 (0.099, 0.115)	0.965	0.114 (0.107, 0.121)	0.006	0.194
*Model 3* ^e^					
Income → ∆ CCS	0.097 (0.086, 0.107)		0.096 (0.087, 0.105)		0.954
CCS → ∆ income	0.097 (0.089, 0.106)	0.884	0.110 (0.103, 0.118)	0.008	0.028
Grip strength (GS)					
*Model 1* ^c^					
Income → ∆ GS	0.087 (0.079, 0.095)		0.066 (0.059, 0.073)		<0.001
GS → ∆ income	0.082 (0.074, 0.089)	0.282	0.080 (0.073, 0.086)	0.003	0.567
*Model 2* ^d^					
Income → ∆ GS	0.053 (0.043, 0.064)		0.025 (0.015, 0.034)		<0.001
GS → ∆ income	0.049 (0.041, 0.058)	0.516	0.040 (0.032, 0.047)	0.007	0.084
*Model 3* ^e^					
Income → ∆ GS	0.046 (0.036, 0.057)		0.023 (0.013, 0.033)		0.002
GS → ∆ income	0.038 (0.030, 0.047)	0.203	0.034 (0.026, 0.041)	0.046	0.249
Quality of life (QoL)					
*Model 1* ^c^					
Income → ∆ QoL	0.161 (0.153, 0.169)		0.161 (0.153, 0.168)		0.986
QoL → ∆ income	0.146 (0.138, 0.154)	0.004	0.141 (0.134, 0.149)	<0.001	0.273
*Model 2* ^d^					
Income → ∆ QoL	0.087 (0.077, 0.098)		0.084 (0.074, 0.094)		0.138
QoL → ∆ income	0.094 (0.086, 0.102)	0.25	0.089 (0.082, 0.096)	0.349	0.236
*Model 3* ^e^					
Income → ∆ QoL	0.078 (0.068, 0.089)		0.080 (0.070, 0.090)		0.804
QoL → ∆ income	0.081 (0.073, 0.089)	0.69	0.083 (0.076, 0.090)	0.565	0.965
Depressive symptoms					
*Model 1* ^c^					
Income → ∆ depression	0.076 (0.069, 0.083)		0.074 (0.068, 0.081)		0.833
Depression →∆ income	0.066 (0.058, 0.073)	0.03	0.069 (0.063, 0.076)	0.211	0.961
*Model 2* ^d^					
Income → ∆ depression	0.053 (0.044, 0.063)		0.046 (0.037, 0.055)		0.574
Depression → ∆ income	0.051 (0.044, 0.058)	0.614	0.050 (0.043, 0.056)	0.494	0.17
*Model 3* ^e^					
Income → ∆ depression	0.042 (0.032, 0.052)		0.042 (0.033, 0.051)		0.985
Depression → ∆ income	0.038 (0.031, 0.045)	0.487	0.044 (0.037, 0.050)	0.711	0.692

^a^*p*-values for differences between the reciprocal associations (i.e., associations between income/wealth and health changes and between health and trajectories of income/wealth). ^b^*p*-values for sex differences within each of the reciprocal associations. ^c^ Model 1: unadjusted. ^d^ Model 2: adjusted for age group, European region, wave, and marital status. ^e^ Model 3: adjusted for age group, European region, wave, marital status, and employment.

**Table 3:** Coefficients and 95% confidence intervals (CIs) for the associations between wealth and health changes (i.e., change (∆) in cognitive function, grip strength, quality of life and depressive symptoms) and for associations between health and trajectories (∆) of wealth.

	Men		Women		Sex Differences (Men vs. Women)
	Coefficients (95% CI)	*p*-Values ^a^	Coefficients (95% CI)	*p*-Values ^a^	*p*-Values ^b^
Cognitive composite score (CCS)					
*Model 1* ^c^					
Wealth → ∆ CCS	0.075 (0.067, 0.082)		0.072 (0.065, 0.079)		0.614
CCS → ∆ wealth	0.078 (0.070, 0.086)	0.479	0.082 (0.075, 0.089)	0.02	0.244
*Model 2* ^d^					
Wealth → ∆ CCS	0.080 (0.071, 0.088)		0.076 (0.069, 0.083)		0.85
CCS → ∆ wealth	0.068 (0.059, 0.077)	0.028	0.069 (0.061, 0.077)	0.124	0.072
*Model 3* ^e^					
Wealth → ∆ CCS	0.070 (0.062, 0.078)		0.074 (0.067, 0.082)		0.76
CCS → ∆ wealth	0.061 (0.052, 0.070)	0.108	0.066 (0.058, 0.074)	0.079	0.183
Grip strength (GS)					
*Model 1* ^c^					
Wealth → ∆ GS	0.054 (0.047, 0.062)		0.047 (0.040, 0.054)		0.184
GS → ∆ wealth	0.072 (0.064, 0.079)	0.001	0.062 (0.055, 0.069)	0.001	0.041
*Model 2* ^d^					
Wealth → ∆ GS	0.060 (0.052, 0.068)		0.040 (0.032, 0.047)		<0.001
GS → ∆ wealth	0.053 (0.044, 0.063)	0.253	0.035 (0.027, 0.043)	0.364	0.018
*Model 3* ^e^					
Wealth → ∆ GS	0.055 (0.047, 0.064)		0.038 (0.030, 0.045)		0.003
GS → ∆ wealth	0.045 (0.036, 0.055)	0.068	0.031 (0.023, 0.039)	0.159	0.004
Quality of life (QoL)					
*Model 1* ^c^					
Wealth → ∆ QoL	0.123 (0.115, 0.131)		0.119 (0.112, 0.127)		0.546
QoL → ∆ wealth	0.101 (0.093, 0.110)	<0.001	0.095 (0.088, 0.102)	<0.001	0.214
*Model 2* ^d^					
Wealth → ∆ QoL	0.090 (0.081, 0.098)		0.091 (0.083, 0.098)		0.221
QoL → ∆ wealth	0.073 (0.064, 0.081)	0.002	0.066 (0.058, 0.074)	<0.001	0.086
*Model 3* ^e^					
Wealth → ∆ QoL	0.083 (0.074, 0.091)		0.088 (0.080, 0.095)		0.486
QoL → ∆ wealth	0.064 (0.055, 0.072)	<0.001	0.061 (0.054, 0.069)	<0.001	0.593
Depressive symptoms					
*Model 1* ^c^					
Wealth → ∆ depression	0.064 (0.056, 0.071)		0.063 (0.057, 0.069)		0.955
Depression → ∆ wealth	0.054 (0.046, 0.061)	0.04	0.051 (0.045, 0.058)	0.005	0.479
*Model 2* ^d^					
Wealth → ∆ depression	0.048 (0.040, 0.056)		0.050 (0.043, 0.057)		0.74
Depression → ∆ wealth	0.036 (0.029, 0.044)	0.018	0.035 (0.028, 0.041)	<0.001	0.07
*Model 3* ^e^					
Wealth → ∆ depression	0.040 (0.032, 0.048)		0.046 (0.039, 0.053)		0.343
Depression → ∆ wealth	0.027 (0.020, 0.035)	0.011	0.030 (0.023, 0.037)	<0.001	0.702

^a^*p*-values for differences between the reciprocal associations (i.e., associations between income/wealth and health changes and between health and trajectories of income/wealth). ^b^*p*-values for sex differences within each of the reciprocal associations. ^c^ Model 1: unadjusted. ^d^ Model 2: adjusted for age group, European region, wave, and marital status. ^e^ Model 3: adjusted for age group, European region, wave, marital status, and employment.

## Data Availability

The SHARE data are available for scientific research after registration at www.share-project.org (accessed on 9 May 2021).

## Supplementary Materials

The following are available online at https://www.mdpi.com/article/10.3390/ijerph18095045/s1. Table S1: Coefficients and 95% confidence intervals (CIs) for the associations between SES (i.e., household income and wealth) and trajectories (∆) of SES, and between health and health trajectories (∆) (i.e., changes in cognitive function, grip strength, quality of life and depressive symptoms).
