# Supplementary material for: The Reciprocal Relationship between Socioeconomic Status and Health and the Influence of Sex: A European SHARE-Analysis Based on Structural Equation Modeling
Source: Int J Environ Res Public Health. 2021 May 10;18(9):5045. doi: 10.3390/ijerph18095045 (PMC8126237; doi:10.3390/ijerph18095045)
Supplement: Supplementary file 1 [file ijerph-18-05045-s001.zip › ijerph-1205841-supplementary.pdf]

Supplementary Table

Table S1. Coefficients and 95% confidence intervals (CIs) for the associations between SES (i.e., income and wealth) and trajectories (Δ) of SES and between health and health trajectories (Δ) (i.e., changes in cognitive function, grip strength, quality of life and depressive symptoms).

	**Men**	**Women**	**Sex diff (men vs women)**[a]
Income	Coefficient (95% CI)	Coefficient (95% CI)	P-values[a]
Cognitive composite score (CCS)			
Income → Δ income	−0.736 (−0.745, −0.728)	−0.786 (−0.794, −0.778)	<0.001
CCS → Δ CCS	−0.510 (−0.518, −0.502)	−0.526 (−0.534, −0.519)	<0.001
Grip strength (GS)			
Income → Δ income	−0.723 (−0.732, −0.714)	−0.772 (−0.781, −0.763)	<0.001
GS → Δ GS	−0.523 (−0.533, −0.513)	−0.525 (−0.535, −0.515)	0.567
Quality of life			
Income →Δ income	−0.738 (−0.748, −0.729)	−0.788 (−0.797, −0.779)	<0.001
Quality of life → Δ quality of life	−0.498 (−0.506, −0.491)	−0.501 (−0.508, −0.494)	0.999
Depressive symptoms			
Income → Δ income	−0.725 (−0.734, −0.717)	−0.774 (−0.782, −0.766)	<0.001
Depression → Δ depression	−0.501 (−0.508, −0.493)	−0.503 (−0.509, −0.497)	0.212
Wealth			
Cognitive composite score (CCS)			
Wealth → Δ wealth	−0.418 (−0.425, −0.411)	−0.426 (−0.432, −0.419)	0.039
CCS → Δ CCS	−0.508 (−0.516, −0.500)	−0.525 (−0.533, −0.518)	<0.001
Grip strength (GS)			
Wealth → Δ wealth	−0.411 (−0.418, −0.403)	−0.416 (−0.423, −0.410)	0.169
GS → Δ GS	−0.526 (−0.536, −0.516)	−0.526 (−0.536, −0.517)	0.653
Quality of life			
Wealth → Δ wealth	−0.424 (−0.431, −0.417)	−0.431 (−0.438, −0.425)	0.093
Quality of life → Δ quality of life	−0.502 (−0.510, −0.495)	−0.506 (−0.512, −0.499)	0.960
Depressive symptoms			
Wealth → Δ wealth	−0.414 (−0.421, −0.407)	−0.421 (−0.427, −0.415)	0.055
Depression → Δ depression	−0.501 (−0.509, −0.494)	−0.504 (−0.510, −0.499)	0.242

[a] *p*-values for sex differences within each of the reciprocal associations; All associations are adjusted for age group, European region, wave, marital status, and employment.
